# Extrapancreatic effects of incretin hormones: evidence for weight‐independent changes in morphological aspects and oxidative status in insulin‐sensitive organs of the obese nondiabetic Zucker rat (ZFR)

**DOI:** 10.14814/phy2.12886

**Published:** 2016-08-10

**Authors:** Ides M. Colin, Henri Colin, Ines Dufour, Charles‐Edouard Gielen, Marie‐Christine Many, Jean Saey, Bernard Knoops, Anne‐Catherine Gérard

**Affiliations:** ^1^ Endocrino‐Diabetology Research UnitCentre Hospitalier Régional (CHR) Mons‐HainautMonsBelgium; ^2^ Faculté de MédecinePôle de Morphologie, Institut de Recherche Expérimentale et Clinique (IREC)Secteur des Sciences de la Santé (SSS)Université catholique de Louvain (UCL)BrusselsBelgium; ^3^ Group of Animal and Molecular Cell BiologyInstitut des Sciences de la VieUniversité catholique de Louvain (UCL)Louvain‐La‐NeuveBelgium

**Keywords:** Exendin‐4, incretin hormones, inflammation, oxidative stress, Zucker rat

## Abstract

Incretin‐based therapies are widely used to treat type 2 diabetes. Although hypoglycemic actions of incretins are mostly due to their insulinotropic/glucagonostatic effects, they may also influence extrapancreatic metabolism. We administered exendin‐4 (Ex‐4), a long‐acting glucagon‐like peptide receptor agonist, at low dose (0.1 nmol/kg/day) for a short period (10 days), in obese nondiabetic *fa/fa* Zucker rats (ZFRs). Ex‐4‐treated ZFRs were compared to vehicle (saline)‐treated ZFRs and vehicle‐ and Ex‐4‐treated lean rats (LRs). Blood glucose levels were measured at days 0, 9, and 10. Ingested food and animal weight were recorded daily. On the day of sacrifice (d10), blood was sampled along with liver, epididymal, subcutaneous, brown adipose, and skeletal muscle tissues from animals fasted for 24 h. Plasma insulin and blood glucose levels, food intake, and body and epididymal fat weight were unchanged, but gross morphological changes were observed in insulin‐sensitive tissues. The average size of hepatocytes was significantly lower in Ex‐4‐treated ZFRs, associated with decreased number and size of lipid droplets and 4‐hydroxy‐2‐nonenal (HNE) staining, a marker of oxidative stress (OS). Myocytes, which were smaller in ZFRs than in LRs, were significantly enlarged and depleted of lipid droplets in Ex‐4‐treated ZFRs. Weak HNE staining was increased by Ex‐4. A similar observation was made in brown adipose tissue, whereas the elevated HNE staining observed in epididymal adipocytes of ZFRs, suggestive of strong OS, was decreased by Ex‐4. These results suggest that incretins by acting on OS in insulin‐sensitive tissues may contribute to weight‐independent improvement in insulin sensitivity.

## Introduction

The incretins, which include glucose‐dependent insulinotropic polypeptide (GIP) and glucagon‐like peptide‐1 (GLP‐1), are intestinal mucosal‐derived hormones released in response to carbohydrates, fat, and proteins contained in food (Campbell and Drucker 2013). Nowadays, incretin‐based therapies are widely used in the treatment of type 2 diabetes (Cernea and Raz 2011; Lund et al. 2014; Mulvihill and Drucker 2014). The best‐known physiological action of GLP‐1 is its ability to stimulate insulin secretion from pancreatic β‐cells in a nutrient‐dependent manner, while inhibiting glucagon secretion (Campbell and Drucker 2013; Drucker 2015). Other activities of GLP‐1 include inhibition of hepatic glucose production, gastric emptying, reduction of food intake and body weight, and regulation of immune functions (Campbell and Drucker 2013; Baggio and Drucker 2014; Secher et al. 2014; Sisley et al. 2014; Drucker 2015). In addition to islet hormone regulation, incretins also influence many other metabolic pathways, by either directly interacting with tissues expressing their receptors or indirectly through neuronal and endocrine pathways (Drucker 2015).

Increasing research effort has been devoted to better understanding the extrapancreatic effects of incretins, especially in insulin‐sensitive organs such as the liver, skeletal muscle cells, and adipose tissues (subcutaneous [SAT], visceral [VAT], and brown [BAT] adipose tissues). This interest has arisen from observations suggesting that in addition to their insulinotropic actions, incretins also influence glucose metabolism by increasing insulin sensitivity in insulin‐targeted organs through mechanisms that remain to be elucidated (Mizuno et al. 1997; Nielsen et al. 2004; Gedulin et al. 2005). The idea that incretins induce anti‐inflammatory and antioxidative effects is receiving growing attention, and merits further exploration (Oeseburg et al. 2010; Chaudhuri et al. 2012; Hendarto et al. 2012; Ceriello et al. 2013, 2014; Batchuluun et al. 2014; Fujita et al. 2014; Buldak et al. 2015; Inoue et al. 2015).

Exenatide (a synthetic version of exendin‐4 [Ex‐4], a long‐acting GLP‐1RA) suppresses production of reactive oxygen species (ROS) and exerts anti‐inflammatory effects at the cellular and molecular levels (Wu et al. 2011; Chaudhuri et al. 2012). Ex‐4 can reverse cardiac dysfunction and steatosis by decreasing cardiac oxidative stress (OS) in a rodent model of type 2 diabetes (Monji et al. 2013). Incretin‐based therapies may also prevent OS in individuals with type 2 diabetes (Ceriello et al. 2013, 2014). Furthermore, independent of changes in body weight and glucose levels, exenatide increases both whole‐body insulin sensitivity and insulin sensitivity‐adjusted β‐cell mass in insulin‐resistant obese nondiabetic *fa/fa* Zucker rats (ZFRs) (Gedulin et al. 2005). These data are consistent with the observation that GLP‐1RA improves hepatic insulin signaling while reducing hepatic fat deposition in animal models of nonalcoholic fatty liver disease (NAFLD) (Ding et al. 2006; Gupta et al. 2010, 2012; Sharma et al. 2011; Lee et al. 2012, 2012, 2014; Mells et al. 2012; Blaslov et al. 2014; Liu et al. 2014; Wang et al. 2014).

In this study, we monitored Ex‐4‐induced morphological changes as well as changes in the oxidative status and lipid contents of insulin‐sensitive organs (liver, skeletal muscles, and adipose tissues) in the ZFR model, a commonly used animal model of obese metabolic syndrome. Because of a defect in hypothalamic leptin signaling due to a natural mutation in the leptin receptor gene (*fa* allele), ZFR are hyperphagic and exhibit early onset of obesity, mild glucose intolerance, insulin resistance, hyperlipidemia, hyperinsulinemia, and moderate hypertension (Kucera and Cervinkova 2014). OS was assessed by the immunodetection of 4‐hydroxynonenal (4‐HNE), an aldehyde product of lipid peroxidation (Spickett 2013). We also looked at the expression of peroxiredoxin 5 (PRDX5), a potent oxidant from the family of peroxiredoxin antioxidant proteins which regulate intracellular levels of ROS (Knoops et al. 1999). As Ex‐4 was administered at low doses (0.1 nmol/kg/day) and for a short period of time (10 days), the reported observations were not confounded by changes in body weight, food intake, or blood insulin and glucose levels.

## Materials and Methods

### Animals and treatments

All procedures were carried out consistent with the regulations and guidelines of the Belgian state and European Union and were approved by the local ethics committee (Permit number: 2011‐2/UCL/MD/024p). All efforts were made to minimize suffering. Six‐week‐old male ZFRs and lean AG2 rats (LR) (Charles River Laboratories, Chatillon‐sur‐Chalaronne, France) were used (eight rats per group, three in the first experiment and five in the second). All rats were fed a normal diet (Scientific Animal Food and Engineering, Augy, France) for 10 days prior to initiation of treatment. ZFRs were then injected once a day intraperitoneally (i.p.) with either Ex‐4 (0.1 nmol/kg/day, Sigma‐Aldrich, Bornem, Belgium) or vehicle (saline) for 10 days. Starting on day 9, rats were fasted for 24 h. Blood glucose was measured on days 0 (the day of the first i.p. injection), 9, and 10. Rats, as well as food, were weighed each day. On day 10, the rats were euthanized using pentothal. Blood was collected, and liver, epididymal fat, white subcutaneous adipose tissue, skeletal muscles (solear), and brown adipose tissue were removed. Epididymal fat, an intra‐abdominal depot that is considered to be representative of visceral fat (VAT), was weighed. Samples of all tissues were fixed in 4% buffered‐formalin and embedded in paraffin, and the remaining portions were frozen in isopentane cooled in liquid nitrogen and used for cryostat sections.

### Blood glucose and serum insulin levels

Blood glucose was measured on days 0, 9, and 10. Following a short‐term anesthesia with isoflurane (few minutes), a drop of blood was collected from the tail, and blood glucose levels were measured using a glucometer (OneTouch, Lifescan, Beerse, Belgium). Serum insulin levels were measured on day 10 using an ELISA kit (Abcam, Cambridge, UK).

### Histology and morphometry

Paraffin‐embedded tissue sections (7‐*μ*m thick) were stained with hematoxylin–eosin, and digital images of stained sections were used for morphometry measurements. Cell surface area of hepatocytes and adipocytes and diameter of skeletal muscle cells were measured using the NIH Scion Image Analysis software (National Institutes of Health, Bethesda, MA). About 500 cells from each tissue were measured in each animal.

### Oil red O staining

Oil red O staining was used to detect intracytoplasmic lipid droplets on liver and muscle sections. Cryostat sections were immediately fixed in 10% formaldehyde and stained with oil red O solution (Sigma‐Aldrich, Bornem, Belgium) for 10 min at RT.

### Immunohistochemistry

4‐hydroxy‐2‐nonenal (HNE) and peroxiredoxin‐5 (PRDX5) were detected in paraffin‐embedded tissue sections (7‐*μ*m thick). For HNE staining, sections were pretreated in citrate buffer (0.01 mol/L) in a microwave oven as described previously (Poncin et al. 2008). All sections were incubated with peroxidase block (DakoCytomation, Heverlee, Belgium) for 5 min at RT, washed with PBS, and incubated with PBS containing 25% nonimmune fetal bovine serum and 10% nonfat dry milk for 45 min at RT. Slides were then incubated with an anti‐HNE rabbit primary polyclonal antibody (1:300) or anti‐PRDX5 rabbit primary polyclonal antibody (1/250) (Gerard et al. 2005) at RT for 1 h, washed with PBS for 10 min, incubated with Envision Flex rabbit LINKER (DakoCytomation) for 15 min at RT, and then incubated with an Envision‐labeled polymer‐HRP anti‐rabbit secondary antibody (DakoCytomation) for 30 min at RT. The peroxidase reaction was visualized by incubating for 5 min with diaminobenzidine (DAB, DakoCytomation) and then counterstained with hematoxylin.

### Statistical analysis

Data for weight, serum insulin levels, and morphometry are expressed as means ± SEM from five animals (*n* = 5), and blood glucose data as means ± SEM from eight animals (*n* = 8). Statistical significance of differences was evaluated using an unpaired or paired Student's *t*‐test. A *P* < 0.05 was considered statistically significant (GraphPad, San Diego, CA).

## Results

### Ex‐4 injected at low doses for a short period of time does not induce changes in body weight, VAT weight, blood glucose, or insulin levels

**Figure 1:**
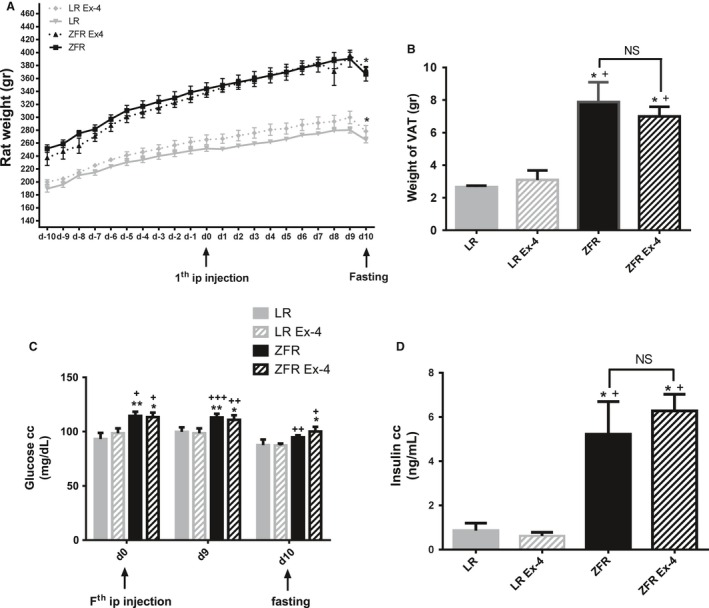
Ex‐4‐induced effects on body weight, VAT weight, blood glucose, and plasma insulin levels. (A) Body weight of ZFRs was significantly higher than that of LRs throughout the experiment. On day 10, after a 24‐h fast, both experimental groups exhibited a significant reduction in body weight. Ex‐4 had no effect on body weight. (B) VAT weight on day 10 was significantly higher in ZFRs than in LRs. Ex‐4 had no effect on VAT weight. (C) Blood glucose levels were significantly higher in ZFRs than in LRs. On day 10, blood glucose values decreased as rats fasted. Ex‐4 had no effect on blood glucose values. (D) Serum insulin levels measured on day 10 were significantly higher in ZFRs than in LRs. Ex‐4 had no effect on serum insulin levels. Results are expressed as means ± SEM, *n* = 5 rats. *P < 0.05; **P < 0.01 versus Lean; ^+^ *P *< 0.05; ^++^ *P *< 0.01; ^+++^ *P *< 0.001 versus Lean+Ex‐4.

The average body weight of ZFRs was significantly higher than that of lean rats (LRs) throughout the experiment (Fig. 1A). Body weights of LRs and ZFRs increased steadily from day 10 (the first day in the animal facility) to day 0, when the first dose of Ex‐4/vehicle was administered. Ex‐4 or vehicle was injected once a day from day 0 to day 9; both groups of animals were perfectly matched in terms of weight and food intake. During this period, body weight continued to increase at the same rate observed during the pretreatment period. No difference was noted between Ex‐4‐treated and vehicle‐treated animals at any time point during this 10‐day period. All animals fasted for 24 h before sacrifice (from day 9 to day 10), and consequently lost weight. The trend was similar in all experimental groups, irrespective of treatment with Ex‐4.

On day 10, animals were sacrificed, and epididymal VAT was removed and weighed. VAT weight was significantly higher in ZFRs than in LRs (Fig. 1B). Ex‐4 treatment did not change VAT weight relative to that in untreated animals. Blood glucose levels were measured on days 0, 9, and 10 (Fig. 1C). Nonfasting blood glucose levels recorded in ZFRs at days 0 and 9, although significantly higher than those in LRs, were not in the diabetic range. Blood glucose levels in fasted ZFRs were significantly lower than those in nonfasted animals, but did not differ significantly from the levels in fasted LRs. Body and VAT weights did not differ between Ex‐4‐ and vehicle‐treated animals. Fasting insulin plasma levels were measured in all experimental groups on day 10 (Fig. 1D). As expected, insulin plasma levels were significantly higher in ZFRs than in LRs. Insulin plasma levels were not affected by Ex‐4 treatment.

### Ex‐4 injected at low doses over a short period of time induces changes in morphological aspects and size of hepatocytes, as well as in HNE and PRDX5 immunohistochemical stainings

**Figure 2:**
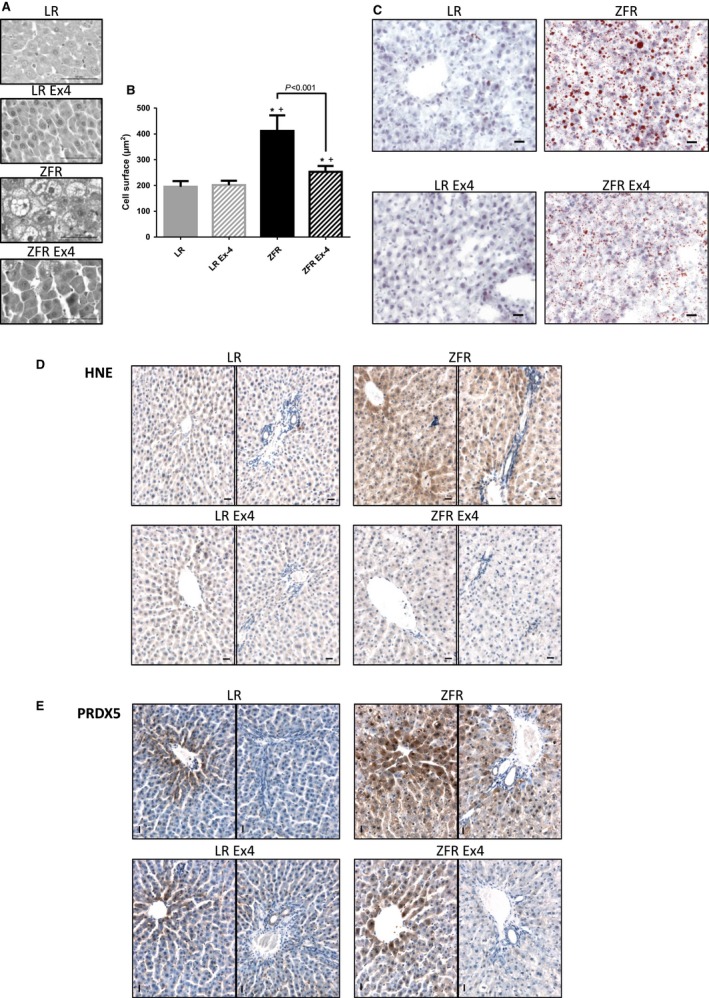
Ex‐4‐induced effects in the liver. (A) Histological aspects of hepatocytes. (B) Cell surface area of hepatocytes. Results are expressed as means (*μ*m^2^) ± SEM, *n* = 5. *P < 0.05 versus LR; ^+^ *P*<0.05 versus LR+Ex‐4. (C) Oil red O staining of lipids. Compared to those of LRs, hepatocytes of ZFRs were enlarged and richer in lipids. Ex‐4 treatment decreased the size of ZFR hepatocytes as well as the size of lipid droplets, but did not induce remarkable changes in LRs. (D) HNE immunostaining. (E) PRDX5 immunostaining. HNE and PRDX5 signals were stronger in ZFRs than in LRs. Ex‐4 treatment reduced both HNE and PRDX5 signals in ZFR. For each condition, a centrilobular (left) and periportal (right) zone are shown. Scale bar = 20 *μ*m.

Compared to those of LRs, hepatocytes of ZFRs were swollen and rich in lipid droplets (Fig. 2A). After 10 days of Ex‐4 treatment, the number and size of lipid droplets were markedly reduced. Ex‐4 treatment significantly decreased the size of ZFR hepatocytes, as determined by morphometry (*P* < 0.05) (Fig. 2B). In contrast, Ex‐4 induced no such changes in LRs (Fig. 2A and B). Numerous oil red O‐stained lipid droplets were observed in ZFRs, but not in LRs (Fig. 2C). Ex‐4 treatment markedly reduced the size (and likely the number) of lipid droplets in ZFRs, suggesting elevated lipolysis (Fig. 2C).

4‐hydroxy‐2‐nonenal staining was stronger in ZFRs than in LRs, indicative of elevated OS in ZFR liver (Fig. 2D). Notably, HNE staining differed regionally within the liver; specifically, it was stronger in cells lining the centrilobular zones than in the periportal zones, suggesting that OS was higher in the centrilobular zones. Ex‐4 treatment markedly reduced HNE staining in ZFRs to a level similar to that in LRs, suggesting that Ex‐4 decreases OS in ZFR hepatocytes.

An immunohistochemical signal for PRDX5 was detected in the centrilobular zones, but not in the periportal zones of LRs (Fig. 2E). In contrast, the PRDX5 signal was present in all areas of the liver in ZFRs, particularly in the centrilobular zones where the HNE signal was also the strongest. PRDX5 was detected in the nuclei of many ZFR hepatocytes. Ten days of Ex‐4 treatment markedly reduced the PRDX5 signal in the centrilobular zones of ZFRs, and to a greater extent in the periportal zones, bringing the signal to a level intermediate between those of untreated ZFRs and LRs. These results indicate that, in parallel with hepatic steatosis, hepatic OS in ZFRs is markedly reduced by Ex‐4.

### Ex‐4 injected at low doses over a short period of time in ZFRs induces changes in morphological aspects and size of skeletal muscle cells, as well as in HNE and PRDX5 immunohistochemical stainings

**Figure 3:**
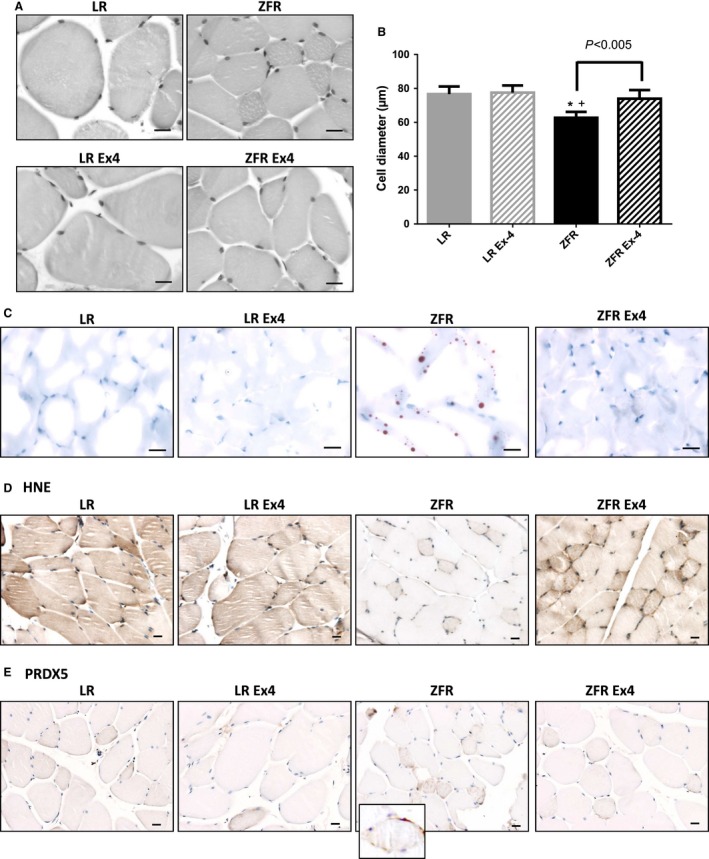
Ex‐4‐induced effects in skeletal muscle cells. (A) Histological aspects of skeletal muscle cells. (B) Cell diameter of myocytes. Results are expressed as means (*μ*m) ± SEM, *n* = 5. *P < 0.05 versus LR; +*P*<0.05 versus LR+Ex‐4. (C) Oil red O staining of lipids. Compared to those in LRs, myocytes of ZFRs were smaller and contained numerous lipid droplets. Ex‐4 treatment increased the size of myocytes in ZFRs. The number of lipid droplets was strongly reduced by Ex‐4. Ex‐4 did not induce remarkable changes in LRs. (D) HNE immunostaining. Diffuse HNE staining in myocytes was observed in LRs, but not in ZFRs, although some small myocytes exhibited strong pericellular staining. Ex‐4 treatment increased HNE staining in all cells. (E) PRDX5 immunostaining. In contrast to LRs, in which no PRDX5 staining was observed, small myocytes of ZFRs exhibited strong immunostaining for PRDX5 (insert). Ex‐4 treatment reduced this pericellular PRDX5 immunostaining. Scale bar = 20 *μ*m.

Morphological (Fig. 3A) and morphometric (Fig. 3B) analyses of skeletal muscle cells showed that myocytes in ZFRs were smaller (*P* < 0.05) and more heterogeneous than those of LRs. After Ex‐4 treatment, ZFR myocytes had a less condensed aspect, and their size increased to approach that of LRs (*P* < 0.005 vs. untreated ZFRs) (Fig. 3B).

As in hepatocytes, oil red O‐stained lipid droplets were observed in ZFRs, but not in LRs, suggestive of elevated intracellular fat deposits (more precisely, intermyofibrillar deposits, as described by Lally et al. (2012) (Fig. 3C). After 10 days of Ex‐4 treatment, oil red O‐stained lipid droplets were no longer observed in ZFR (Fig. 3C).

In contrast to hepatocytes, overall diffuse myocyte HNE staining was weaker in ZFRs than in LRs, although strong pericellular staining was observed in the smallest cells (arrow, Fig. 3D). This observation suggests a global reduction in oxidative phosphorylation in the healthiest cells and elevation of OS in the smallest (i.e., most altered) cells. Following Ex‐4 treatment, HNE staining was elevated in all cells (even the smallest ones), suggesting that Ex‐4 treatment restored oxidative phosphorylation activity in myocytes of ZFRs (Fig. 3D).

In contrast to LRs, in which no remarkable changes were observed between Ex‐4‐ and vehicle‐treated animals, PRDX5 expression in ZFRs was higher in the small myocytes with the strongest HNE staining (Fig. 3E). Following Ex‐4 treatment, PRDX5 staining in these small cells decreased to a level similar to that in LRs (Fig. 3E). These changes suggest that, in addition to increasing the level of oxidative phosphorylation in ZFR myocytes, Ex‐4 also decreases the level of potentially harmful OS in the most affected myocytes.

### Ex‐4 injected at low doses over a short period of time in ZFRs induces changes in morphological aspects and size of VAT adipocytes, as well as in the number of HNE‐positive cells

**Figure 4:**
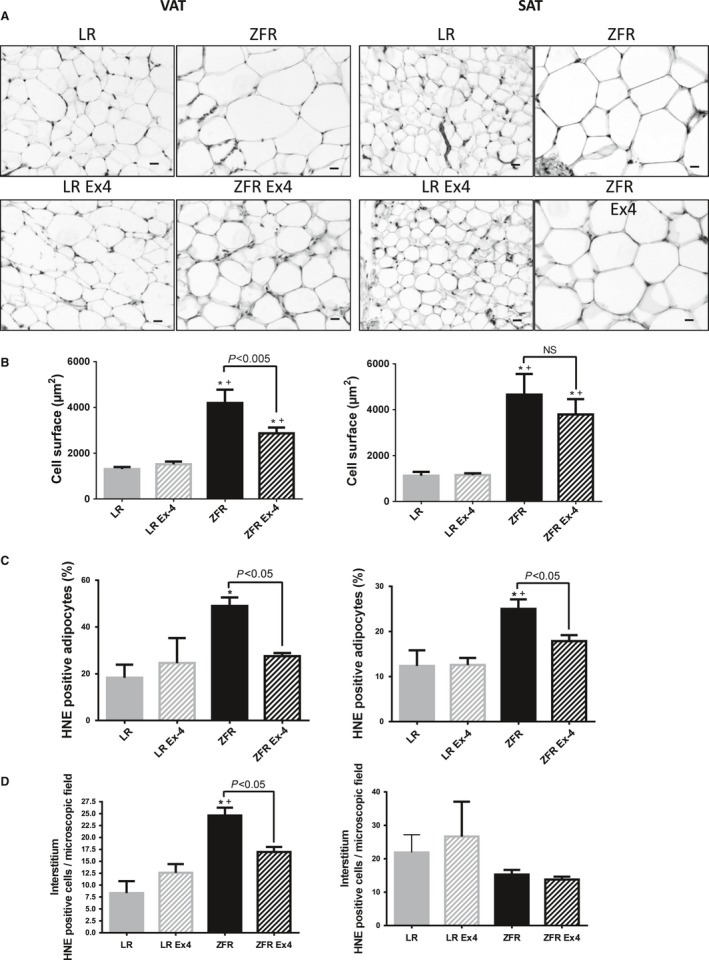
Ex‐4‐induced effects in VAT and SAT. (A) Histological aspects of VAT (left) and SAT (right). In both VAT and SAT of ZFRs, the adipocytes were significantly larger than in LRs. Inflammatory cells were observed in the interstitium of VAT. (B) Cell surface are of adipocytes (*μ*m^2^) in VAT (left) and SAT (right). Following Ex‐4 treatment in ZFRs, the size of VAT adipocytes was reduced. (C) Number of HNE‐positive adipocytes in VAT (left) and SAT (right). (D) Number of HNE‐positive interstitial cells in VAT (left) and SAT (right). The number of VAT HNE‐positive adipocytes and interstitial cells was higher in ZFRs than in LRs. Following Ex‐4 treatment in ZFRs, the number of VAT HNE‐positive adipocytes and interstitial cells significantly decreased, as did the number of SAT HNE‐positive adipocytes. Results are expressed as means ± SEM, *n* = 5. *P < 0.05 versus LR; ^+^ *P* < 0.05 versus LR+Ex‐4. Scale bar = 20 *μ*m.

The cell surface area of VAT and SAT adipocytes was significantly higher in ZFRs than in LRs (*P*<0.005) (Fig. 4A and B). Numerous infiltrating inflammatory cells were present in VAT interstitium, but not in SAT (Fig. 4A). Following Ex‐4 treatment in ZFRs, the cell surface area of VAT adipocytes was significantly reduced (*P *< 0.002), suggestive of elevated lipolysis (Fig. 4B).

The number of VAT HNE‐positive adipocytes and interstitial cells was significantly higher in ZFRs than in LRs (47.25% increase; *P *< 0.05) (Fig. 4C and D), as was the number of SAT HNE‐positive adipocytes (25% increase; *P *< 0.05) (Fig. 4C). The number of SAT HNE‐positive interstitial cells did not differ between ZFRs and LRs (Fig. 4D). Following Ex‐4 treatment in ZFRs, the number of VAT HNE‐positive adipocytes and interstitial cells was significantly reduced (Fig. 4C and D), as was the number of SAT HNE‐positive adipocytes (Fig. 4C).

### Ex‐4 injected at low doses over a short period of time in ZFRs induces changes in morphological aspects and size of BAT adipocytes, as well as in HNE and PRDX5 immunohistochemical stainings

**Figure 5:**
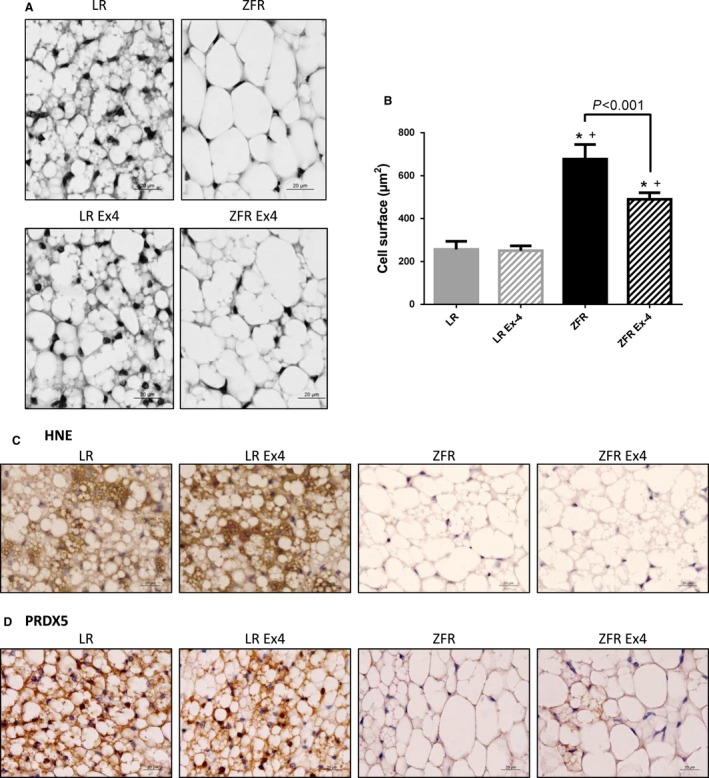
Ex‐4‐induced effects in BAT. (A) Histological aspect of BAT. Ex‐4 partially restored the multilocular aspect of BAT adipocytes in ZFRs. (B) Cell surface area of BAT adipocytes (*μ*m^2^). The increased size of BAT adipocytes observed in ZFRs was significantly decreased by Ex‐4 treatment (*P* < 0.001). Results are expressed as means ± SEM, *n* = 5. *P<0.05 versus LR; +*P*<0.05 versus LR+Ex‐4. (C) HNE immunostaining. (D) PRDX5 immunostaining. Both HNE and PRDX5 signals were strong in BAT of LRs, but almost absent in ZFRs. Ex‐4 had no effect on HNE staining. Ex‐4 increased the PRDX5 staining to some extent in BAT adipocytes of ZFRs, but only in areas where the multilocular aspect was partially restored. Scale bar = 20 *μ*m.

Cell surface in BAT was significantly higher in ZFRs than in LRs (*P *< 0.05) (Fig. 5A and B). The typical multilocular aspect of BAT observed in LRs was lost in ZFRs, but was partially restored following Ex‐4 treatment (Fig. 5A). The cell surface area of BAT adipocytes was also significantly reduced in Ex‐4‐treated ZFRs (*P *< 0.001) (Fig. 5B).

4‐hydroxy‐2‐nonenal staining in BAT was markedly lower in ZFRs than in LRs, suggestive of alterations in oxidative phosphorylation as observed in myocytes (Fig. 5C). Following Ex‐4 treatment in ZFRs, HNE staining in BAT was not restored (Fig. 5C). PRDX5 staining of BAT was stronger in LRs than in ZFRs (Fig. 5D). Following Ex‐4 treatment, the PRDX5 signal in BAT remained weaker in ZFRs, except in areas where the multilocular aspect was partially restored (Fig. 5D).

## Discussion

Our data show that even when administered at low doses (0.1 nmol/kg/day) over a short period of time (10 days), Ex‐4 induces rapid, weight‐independent, and significant morphological changes in insulin‐sensitive organs of ZFRs, including alterations in cell size, OS, and ectopic fat deposits. Based on these findings and previously published data, we propose that the GLP‐1RA‐induced reduction in cellular OS accounts, at least in part, for rapid incretin‐induced improved insulin sensitivity and metabolic health in insulin‐sensitive organs, independent of insulinotropic effects and changes in food intake or body weight.

Some concerns concerning the specificity of the Ex‐4‐induced effects could be raised because of the long fasting period (24 h) before the sacrifice. The length of this period was chosen (instead of a shorter overnight fasting) to ensure that all animals were in the exact same experimental conditions considering, for instance, differences in the stomach repletion between ZFR and LR. To make sure that this long fasting period was not per se responsible for the reported observations, four different experimental groups were considered (ZFR with/wo Ex‐4 and LR with/wo Ex‐4). The strong differences observed between LR and ZFR despite the 24 h‐long fasting period contribute to consider the ZFR model as valid. Differences observed between Ex‐4‐treated and untreated animals (especially ZFR) are therefore really due to GLP‐1RA‐induced effects, and not to those resulting hypothetically from the fasting state.

Until recently, research on GLP‐1RA mostly focused on its pancreatic effects. In addition to glucose‐dependent insulinotropic/glucagonostatic effects, GLP‐1R activation promotes cellular pathways involved in cell survival, inhibition of apoptosis, and the β‐cell stress response (Campbell and Drucker 2013; Drucker 2015). Bearing in mind that GLP‐1Rs are also expressed in many extrapancreatic tissues (Wei and Mojsov 1995; Korner et al. 2007; Gupta et al. 2010), and notwithstanding the controversies associated with this claim (Campbell and Drucker 2013; Drucker 2015), it is reasonable to postulate the existence of additional mechanisms responsible for the recently identified positive effects of incretins in the cardiovascular, immune, and central nervous systems (Campbell and Drucker 2013). Nevertheless, the mechanisms underlying the effects of incretins on insulin resistance remain poorly understood.

In the liver, a large reduction in the size of lipid droplets and a reduction in OS were observed following Ex‐4 treatment, suggesting elevated lipolysis, reduced steatosis, and improved insulin sensitivity. Steatosis in ZFRs is often used as a model of NAFLD, although this system does not advance to nonalcoholic steatohepatitis (NASH) because the second “hit” for the progression from steatosis to steatohepatitis (e.g., exposure to lipopolysaccharides) is absent (Kucera and Cervinkova 2014). Because the redox status of ZFRs is characterized by low amounts of glutathione (GSH) and vitamin E and reduced catalase activity, elevated cellular OS is often observed in these animals (Soltys et al. 2001). In this study, we detected elevated OS in hepatocytes, as reflected by the level of 4‐HNE. The 4‐HNE signal differed regionally and was stronger in the centrilobular regions. Short‐term treatment with Ex‐4 significantly decreased 4‐HNE staining, suggesting that this compound exerts a rapid effect on OS. PRDX5 staining followed the same trend, suggesting that elevated expression of potent antioxidant systems plays a less important role in Ex‐4‐induced OS reduction. These results suggest that the GLP‐1RA‐induced restoration of redox balance in ZFR hepatocytes occurs early in the cascade of cellular and biochemical events that lead to reduced steatosis. Thus, as already suggested (Liu et al. 2014), one mode of action of incretins at the hepatocyte level could be a marked and rapid reduction in OS, associated with elevated lipolysis and restoration of balance between hepatic lipid intake, synthesis, degradation, and secretion. These results are in accordance with previously reported findings. For instance, when *ob/ob* mice (another leptin‐deficient animal model) are treated with Ex‐4, they exhibit a significant decrease in hepatic levels of thiobarbituric reactive substance, a marker of lipid peroxidation (Ding et al. 2006).

Even though Ex‐4 was recently shown to reduce hepatic steatosis through activation of SIRT1 (Lee et al. 2012, 2012, 2014), it remains unclear how GLP‐1RA can restore redox balance in hepatocytes so rapidly. In addition to reducing OS after short‐term exposure, and among many other effects on liver steatosis, GLP‐1RA activation over longer periods of time influences the expression of many genes involved in hepatic lipid metabolism, as well as endoplasmic reticulum stress markers such as GRP78 and C/EBP (Blaslov et al. 2014; Liu et al. 2014; Wang et al. 2014).

In contrast to hepatocytes, skeletal muscle cells were significantly larger in Ex‐4‐treated ZFRs than in vehicle‐treated ZFRs. However, the lipid content in these cells, as determined by the oil red O staining, was markedly decreased by Ex‐4 treatment, suggesting an elevation of Ex‐4‐induced lipolysis and reduction in ectopic fat depots, as observed in hepatocytes. Diffuse 4‐HNE labeling, which was barely detectable in myocytes of vehicle‐treated ZFRs, was elevated following Ex‐4 treatment. In addition, the strong HNE signal present at the periphery of the smallest cells (likely the most affected ones) tended to decrease after Ex‐4 treatment, suggesting reduced ROS production in these cells.

In skeletal muscle, mitochondrial uncoupling and defects in mitochondrial oxidative phosphorylation and ATP synthesis are associated with insulin‐resistant states (Abdul‐Ghani and DeFronzo 2010; Pagel‐Langenickel et al. 2010). Because these defects are associated with elevated intermyofibrillar fat content due to a decrease in mitochondrial fatty acid oxidation (Kelley et al. 2002; Ritov et al. 2005; Lally et al. 2012), it is tempting to speculate that along with improving mitochondrial oxidative and coupling capacities and in turn increasing insulin sensitivity, Ex‐4 might also increase fat clearance from myocytes, further contributing to insulin sensitivity. This could explain the apparently contradictory increase in the 4‐HNE signal in myocytes, which from this standpoint would in fact reflect improved mitochondrial bioenergetics. Thus, in vehicle‐treated ZFRs, mitochondria might be weakly functional but produce relatively low levels of harmful ROS. Consistent with this, Conti et al. showed that the mitochondrial antioxidant defense system is preserved in ZFRs (Conti et al. 2004). This study was not designed to directly address questions related to mitochondria defects. Therefore, the possibility of reactivation of the respiratory chain of oxidative phosphorylation should be tested in future studies. Nevertheless, our results are consistent with those published by Monji et al. who showed Ex‐4‐induced reduction in OS in cardiomyocytes in mouse models of both genetic and diet‐induced obesity. Those authors found that Ex‐4 decreased OS in diabetic cardiomyocytes by suppressing NADPH oxidase 4 (NOX4) and restoring expression of antioxidants such as superoxide dismutase (SOD)‐1 and glutathione peroxidase (Monji et al. 2013).

Obesity in ZFR is characterized by increased visceral fat mass, altered adipose tissue function, changes in extracellular matrix, cellular stress responses, infiltration of immune cells such as macrophages, and chronic inflammation (Kucera and Cervinkova 2014). We observed decreased local microinflammation in VAT following Ex‐4 treatment, and the 4‐HNE level was also markedly reduced under this condition. In contrast, no such changes were observed in SAT. These data are consistent with observations that GLP‐1RA induces lipolysis in 3T3‐L1 adipocytes, decreases macrophage infiltration, and improves in insulin sensitivity (Vendrell et al. 2011; Lee et al. 2012). However, our findings do not exclude indirect effects through increased sympathetic outflow, as recently proposed (Campbell and Drucker 2013). In BAT, even though the size of lipid droplets decreased along with partial restoration of the multilocular aspect, HNE staining was not increased, suggesting that oxidative capacities were not restored, as suggested in myocytes. Whether the effects of Ex‐4 in BAT are direct or indirect (i.e., mediated via the central nervous system) should be addressed in future investigations.

The data presented in this article are consistent with findings published by Gedulin et al. (2005). In ZFRs treated for 6 weeks with Ex‐4 treatment, those authors observed an improvement in the insulin sensitivity index as well as elevated β‐cell mass. HbA1c, fasting glucose, fasting insulin, and daily food consumption were comparable between Ex‐4‐ and pair‐fed vehicle‐treated animals. Collectively, these data support a link between GLP‐1RA activation, OS reduction in insulin‐sensitive tissues, and overall reduction in insulin resistance.

In summary, our data indicate that beyond its well‐known insulinotropic effects, GLP‐1RA, even administered at low doses over short periods, induces rapid changes in insulin‐sensitive tissues. Although the underlying mechanisms remain to be elucidated, it is clear that Ex‐4 decreases OS in hepatocytes while reducing steatosis, and improves perhaps oxidative efficacy in myocytes while decreasing intermyofibrillar fat depots. In addition, Ex‐4 decreases OS and inflammatory infiltration in VAT. Thus, Ex‐4 exerts a dual effect: While it acts as antioxidant modulator to prevent inappropriate ROS production, it could also improve natural oxidative capacities in insulin‐sensitive tissues. Thus, in addition to its combined insulinotropic/glucagonostatic effects, Ex‐4 could contribute to restoration of glucoregulation by improving insulin sensitivity. Although attractive, these data obtained from an animal model should be cautiously extrapolated to the human.

## Conflict of Interest

The authors declare that no conflicts of interest exist.
